# miR‐640 aggravates intervertebral disc degeneration via NF‐κB and WNT signalling pathway

**DOI:** 10.1111/cpr.12664

**Published:** 2019-07-25

**Authors:** Wengang Dong, Jun Liu, Yang Lv, Fei Wang, Tao Liu, Siguo Sun, Bo Liao, Zhen Shu, Jixian Qian

**Affiliations:** ^1^ Department of Orthopaedics The Second Affiliated Hospital of Air Force Medical University Xi’an China; ^2^ Department of Orthopaedics General Hospital of Lanzhou Military Command Lanzhou China; ^3^ Laboratory of Molecular Biology Disease Control and Prevention Center of PLA’s Southern Theatre Command Guangzhou China; ^4^ Biotechnology Center, School of Pharmacy Air Force Medical University Xi’an China; ^5^ Department of Ophthalmology Eye Institute of China PLA, The First Affiliated Hospital of Air Force Medical University Xi’an China; ^6^ Department of Radiation Oncology Winship Cancer Institute, Emory University School of Medicine Atlanta GA USA

**Keywords:** inflammation, intervertebral disc degeneration, microRNA, nucleus pulposus, WNT signalling pathway

## Abstract

**Objectives:**

Low back pain becomes a common orthopaedic disease today. It is mainly induced by the degeneration of the intervertebral disc. In this study, we tried to reveal the pathogenesis of the degeneration and the relative therapeutic strategy, which are still elusive.

**Materials and Methods:**

We collected 15 degenerative intervertebral tissues and five healthy donors. Nucleus pulposus and annulus fibrosus cells were subcultured. miR‐640 expression was determined by qPCR. Computer analysis and luciferase reporter assay were used to confirm miR‐640 target genes. Immunohistochemical and immunocytochemical staining was used to trace the proinflammatory cytokines and key transductor of signalling pathways. We also used β‐galactosidase staining, flow cytometry, and cell viability assay to monitor the degenerative index.

**Results:**

miR‐640 overexpressed in patients derived degenerative nucleus pulposus tissues and cells. The inflammatory environment promoted miR‐640 expression via NF‐κB signalling pathway. In addition, miR‐640 targeted to LRP1 and enhances NF‐κB signal activity, which built a positive feedback loop. miR‐640 inhibited the expression of β‐catenin and EP300, therefore, restrained WNT signal and induced the degeneration in nucleus pulposus cells. miR‐640 inhibitor treatment exhibited the effects of anti‐inflammation, reverse WNT signalling pathway exhaustion, and remission of degenerative characteristics in vitro.

**Conclusions:**

miR‐640 plays an important role in the degeneration of intervertebral disc and the relative inflammatory microenvironment. It is a promising potential therapeutic target for the low back pain biotherapy.

## 1 INTRODUCTION

Low back pain is a global challenge that causes severe health and socioeconomic burdens.^1^, ^2^ Nearly 80% of people will suffer from it in their own life span.^3^ The current aetiology attributes the pain to the intervertebral disc degeneration (IDD).^4^ The intervertebral disc (IVD) is composed of the peripheral annulus fibrosus (AF) and the internal nucleus pulposus (NP). The degeneration mainly displays as a large number of the NP cell apoptosis and the extracellular matrix remodeling.^5^ Although mechanical compression, senescence, genetics, autoimmune, and toxicant^6^, ^7^, ^8^ have been demonstrated to induce IDD in different animal models, the cell events and the underlying mechanisms of IDD pathogenesis are still elusive.^9^

In recent years, many signalling pathways have been found to be associated with IDD.^8^ The canonical NF‐κB signal, which is widely activated in immune reactions, tumour microenvironment, ageing, and cell stress responses,^10^ plays an important role in IDD development as well. Under TNF‐α,^11^ IL‐1,^12^ and hypoxia^13^ stimulation, NF‐κB activates and promotes matrix metalloproteinases (MMPs), proinflammation cytokines (ADAMTS‐5), FasL, and Bax expression. Consequently, it aggravates the inflammatory circumstance and NP cell apoptosis. WNT‐β‐catenin is another IDD‐relevant signalling pathway.^8^ During the development of intervertebral disc, the WNT signal is strictly controlled in the entire process.^14^ The ectopic expression of WNT‐associated signal transductors leads to development disordered, growth plate fracture, and fibrosis tissue overgrowth.^14^ In the early stage of IDD, the reduced WNT signal induces the NP cells to transdifferentiate into chondroid‐like cells and, as a consequence, inhibits NP cell proliferation.^15^ In contrast, the overexpression of WNT11 promotes NP cell differentiation from adipose‐derived stem cells. Therefore, the regulation of WNT signal pathway may be an option for IDD treatment.^16^ Furthermore, a few studies demonstrate that NF‐κB and WNT signalling pathways have multilayered collaboration in diverse physiological and pathological backgrounds. In breast cancer cells, β‐catenin binds to p65‐p50 complex and inhibits its nuclear translocation.^17^ β‐TrCP1 simultaneously activates NF‐κB and inhibits WNT pathways in the vascular smooth muscle cells.^18^ IKKα inhibitor blocks WNT downstream gene *CCND1* expression in mouse embryo fibroblasts.^19^ These findings imply that the crosstalk of the two pathways should be the key to IDD target treatment.

Accumulating evidence shows that the aberrant miRNA level is associated with various aspects of IDD,^20^ such as NP cell apoptosis,^21^ proliferation,^22^ the extracellular matrix regeneration^23^ and inflammatory response.^24^ In this study, based on the previous work,^25^ we noticed that miR‐640 is dramatically up‐regulated in IDD tissues. The analysis of bioinformatics suggested that it probably involves NF‐κB and WNT signalling pathway. Therefore, we planned to reveal its functions and the underlying mechanisms in IDD‐associated inflammatory environment and the regulatory networks. Thus, it may provide a novel biotherapeutic strategy for IDD.

## 2 METHODS AND MATERIALS

### 2.1 Ethics statement

All of the experimental protocols were approved by the Clinical Research Ethics Committee of the Second Affiliated Hospital of Air Force Medical University. Human NP specimens were obtained from patients undergoing discectomy following approval from the Clinical Research Ethics Committee of the Second Affiliated Hospital, with fully informed, written consent from the patients.

### 2.2 Sample collection (patients and samples)

Human NP specimens were collected from patients with idiopathic scoliosis as control (n = 8; average age 24.125, range 18‐33 years) and from patients with IDD (n = 15; average age 34.6, range 27‐46 years, Table S2). Human NP specimens were classified as grade II (idiopathic scoliosis discs) and grade IV (IDD discs) according to MRI. The degree of disc degeneration was graded by using Pfirrmann classification. One normal NP tissue, which is embedded in paraffin and used in the immunohistochemical assay, was stored in our laboratory.

### 2.3 Cell culture and transfection

Human NP tissues were obtained upon microendoscopic discectomy or scoliosis surgery from Second Affiliated Hospital of Air Force Medical University. The tissue specimens were first washed with PBS until clean, and the NP was separated from the AF using a stereotaxic microscope, cut into pieces (2‐3 mm^3^), and the NP cells were released from the NP tissues by incubation with 0.25 mg/mL type II collagenase (Gibco, 17101015) for 6 hours at 37°C in DMEM‐F12 (Gibco, 11320‐033). The digest was filtered through a 45 µm pore size nylon mesh （Millipore, Z290793‐100EA）. Cells were plated in a 6 cm culture dish and expanded for 3 weeks in DMEM/F12‐based culture medium, containing 15% foetal bovine serum (FBS, Gibco, 10099141) and 1% penicillin/streptomycin (Invitrogen, 15070063) in a 37°C, 5% CO_2_ (*v*/*v*) incubator. The culture medium was changed every 72 hours. Primarily cultured NP cells (passage 2‐5) were used for subsequent experiments of miR‐640. 293T cell was cultured in DMEM (Gibco, 11965‐092), containing 10% FBS and 1% penicillin/streptomycin in a 37°C, 5% CO_2_ (*v*/*v*) incubator. The cells were treated by 10 ng/mL TNF‐α (R&D, 210‐TA‐020) for 2 hours, 10 ng/mL IL‐1β (R&D, 201‐LB‐010) for 2 hours, 20 ng/mL cycloheximide (CHX, R&D, 0970/100) for 1 hour, 20 µmol/L Bay11‐7082 (Selleckchem, S2913) for 2 hours, 20 µmol/L SP600125 (Selleckchem, S1460) for 2 hours, 20 µmol/L SB203580 (Selleckchem, S1076) for 2 hours, 100 ng/mL DKK1 (R&D, 5439‐DK‐010) for 4 hours, and 1 µg/mL LPS (Sigma, L2654) for 1 hour in the specific assays. We used Lipofectamine 3000 (Invitrogen, L3000015) to perform the indicated transfection (The miR‐640 mimics and inhibitors were synthesized by RiboBio. p65siRNA, Santa Cruz, sc‐29410; LRP1siRNA, Santa Cruz, sc‐40101).

### 2.4 RNA isolation and real‐time PCR

Total RNA was extracted from harvested cells and tissues with TRIzol (Invitrogen, 15596018), according to the instruction. Then, cDNA was reversely transcribed by using TaqMan® MicroRNA Reverse Transcription Kit (Invitrogen, 4366597), or PrimeScript® 1st Strand cDNA Synthesis Kit (Takara, 6110A). qPCR was performed to measure the indicated gene expression by using TaqMan® 2 × Universal PCR Master Mix (Invitrogen, 4352042): denaturation 95°C, 10 min; 40 cycles of 95°C for 15 seconds, 60°C for 1 minute and 72°C for 45 seconds. The expression of mature miR‐640 was normalized to U6 mRNA. Target gene expression was normalized to β‐actin mRNA. The final data were operated by 2^−ΔΔCt^ to obtain the relative variation. All the primers were listed in Table S1.

### 2.5 H&E and immunohistochemical (IHC) staining

The collected NP tissues from both normal and IDD patients were fixed in formalin and embedded in paraffin. The slices were immersed into xylene and gradient concentration of ethanol solutions. For H&E staining, we dye the slices with eosin, acid alcohol, and haematoxylin. For IHC staining, after antigen retrieval and goat serum blockade, we added TNF‐α antibody (Abcam, ab1793, 1:50) onto the slices and incubated at 4°C overnight. Then, the slices were hybridized with HRP‐labelled secondary antibody. Afterwards, we stained the samples by diaminobenzidine (DAB) and haematoxylin. All slices were immersed into xylene and gradient concentration of ethanol solutions again. The images were taken on a microscope (Olympus, IX‐71) with a 20× objective lens.

### 2.6 Western blot

Different treated NP cells were completely lysed in ice‐cold RIPA buffer. Nuclear protein was extracted by using NE‐PER Nuclear and Cytoplasmic Extraction Reagents (Thermo, 78833). Proteins were separated in a 10% SDS‐PAGE gel and then transferred to PVDF membranes. The membranes were subsequently blocked with 3% BSA for 2 hours and then incubated with LRP1 (Cell Signaling, 64099, 1:1000), EP300 (Cell Signaling, 86377, 1:1000), β‐Catenin (Cell Signaling, 8480, 1:1000), Histone H3 (Cell Signaling, 9728, 1:1000) and β‐actin (Santa Cruz, 376421, 1:200) primary antibodies overnight at 4°C and HRP‐conjugated secondary antibodies. The immunoreactive proteins were visualized through enhanced chemiluminescence. The quantification of bands was performed on Image J.

### 2.7 Plasmids construction and dual‐luciferase reporter assays

For NF‐κB regulates miR‐640 expression assay, we synthesized the binding sites and the mutant DNA fragments. After annealing, the dsDNAs were inserted into the luciferase vector pGL3‐basic (Promega, E1751). For miR640 target gene identification assays, we synthesized the complementary rc‐miR640 and the predicted target sequence in *LRP1*, *CTNNB1* and *EP300* genes 3’ UTR. Then, the oligomers were annealed and inserted into psiCHECK vector (Promega, C8011). For luciferase reporter assays, HEK‐293T cells were planted in 6‐well plates (1‐3 × 10^6^/well) and transfected with 0.8 μg of the recombinant plasmids, either alone or in combination with 30 nmol/L miR‐640 precursors or inhibitors. 0.8 μg of pRL vector (Promega, E2231) was transfected to each well simultaneously. After 24 hours, we measured firefly luciferase (FLuc) and renilla luciferase (RLuc) activities by using the Dual‐Luciferase® Reporter Assay System (Promega, E1910).

### 2.8 Immunofluorescence

Coverslips were placed into 24‐well plates, and NP cells were planted on it. Then, the medium was removed and cells were washed twice with cold PBS. Then, cells were fixed in 4% paraformaldehyde for 10 minutes and washed with PBS three times and permeabilized with 0.1% Triton X‐100 in PBS for 15 minutes. Subsequently, the cells were blocked with 1% goat serum for 1 hour, followed by incubation with a rabbit monoclonal primary antibody against p65 (Cell Signaling, 8242, 1:100) and β‐Catenin (Cell Signaling, 8480, 1:100) at 4°C overnight. After washing with PBS, cells were treated with Alexa 488‐conjugated secondary antibody (Thermo, R37114) for 30 minutes and then counterstained with DAPI for 5 minutes to label the cell nuclei. Finally, the images were taken on the FV1000 confocal microscope (Olympus) with 40× objective lens. The fluorescent intensity was measured by using Image J.

### 2.9 Chromatin immunoprecipitation (ChIP)

ChIP was performed according to the manufacturer's protocol of the Chromatin Immunoprecipitation (ChIP) Assay Kit (Millipore, 17‐295). HEK293T cells were transfected with p65 (p65 cDNA was cloned in pcDNA3.1 + vector) or p65 siRNA and then fixed with 1% formaldehyde and incubated with modest shaking for 30 minutes at room temperature. The pellets were resuspended and lysed, and the cells were washed twice with cold PBS. Next, nuclei were isolated and sonicated to produce DNA fragments. Bind anti‐p65 antibodies specific to the DNA‐binding protein to isolate the complex by precipitation. Control IgG was also used for the ChIP assays. The precipitated DNA fragments were finally detected by real‐time PCR and normalized against the input.

### 2.10 β‐Galactosidase Staining

We used the Senescence Cells Histochemical Staining Kit (Sigma, CS0030) to detect ageing cells. NP cells were seeded in 6‐well plates in triplicate (8 × 10^3^/well). The medium was removed, and cells were washed twice with PBS. NP cells were fixed in stain‐fixative for 15 minutes at room temperature and then washed three times in PBS and incubated with dyeing working liquid in a 37°C, 5% CO_2_ (*v*/*v*) environment overnight. Next day, images were collected via a microscope (Olympus, IX‐71). The positive cell was quantified by Image J.

### 2.11 Flow cytometry (FCM)

NP cells were stimulated with TNF‐α and DKK1, either alone or in combination with the transfection of miR‐640 mimics or a miR‐640 inhibitor, and then cultured at 37°C with 5% CO_2_ in humid air. The cells were washed twice with PBS and centrifuged. For apoptosis detection, discarded the supernatants and the cells were resuspended in 1 × annexin‐binding buffer. The apoptosis rate was detected by staining with Annexin V‐FITC and propidium iodide (Millipore, APOAF‐50TST). For cell cycle analysis, the cells were fixed in 70% ethanol overnight and underwent propidium iodide staining (Thermo, F10797). Finally, all samples were detected on FC500 flow cytometry (Beckman).

### 2.12 ELISA

We planted NP cells into the 6‐well plates (1‐3 × 10^6^/well) and transfected the cells with miR‐640 or LRP1 siRNA. LPS was added as a positive control. After 24 hours, the media were collected and undergone the detection of TNF‐α and IL‐1β concentration by using ELISA Kit (R&D, DTA00D, DLB50).

### 2.13 Cell viability

We planted NP cells into the 6‐well plates (1‐3 × 10^6^/well) and transfected the cells with miR‐640. After 24 hours, we digested the cells and re‐planted them into the lightproof 96‐well plates (3 × 10^3^/well). TNF‐α and DKK1 were added every 24 hours. Then, the cell viability was measured by using the CellTiter‐Glo® Luminescent Cell Viability Assay (Promega, G7570) at the indicated time points.

### 2.14 Statistical analysis

Data from all experiments are reported as the mean ± SD from at least 3 independent experiments. All the data were analysed with two‐tailed Student's *t* test, one‐way ANOVA or Mann‐Whitney test by GraphPad Prism 7. When *P* < .05, the differences were considered statistically significant.

## 3 RESULTS

### 3.1 miR‐640 is overexpressed in degenerated NP cells

**Figure 1:**
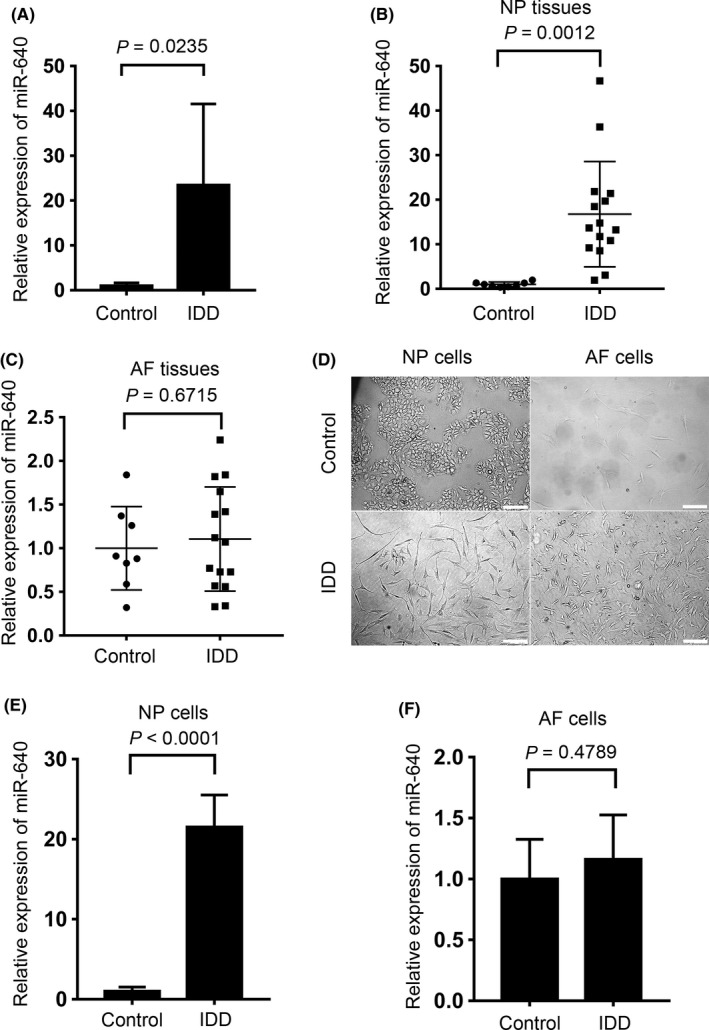
miR‐640 overexpressed in the degenerated NP tissues and cells. A, The microRNA array screening showed the differential expression of miR‐640 in control and IDD tissues. B,C, The levels of miR‐640 in NP and AF tissues were compared between control and IDD tissues, respectively, by qPCR. D, The normal and degenerated IVD derived, primary cultured NP and AF cells. Scar bar, 200 µm. E,F, miR‐640 expression levels were detected in NP and AF primary cells by qPCR. Unpaired Student's *t* test was used to estimate the significant difference for all the qPCR data

Although accumulating evidence indicates that microRNA plays an important role in many pathological processes, its functions in IDD development are still not clear. Therefore, our colleagues Wang et al compared the global microRNA expression between the IDD and the idiopathic scoliosis NP cells by using microRNA array.^25^ The data were submitted to GEO database (GSE19943). They found that 29 miRNAs expression significantly changed in IDD NP cells among 678 human miRNAs. In that study, miR‐640 was the most varied gene and it highly expressed in the degenerated IVD (Figure 1A). To confirm this, we detected miR‐640 levels in 23 IDD and control samples (idiopathic scoliosis). We found that the enhanced expression of miR‐640 only occurred in NP tissues, but not in AF tissues (Figure 1B,C). Then, we separated and subcultured NP and AF cells from control and IDD samples, respectively. A definite mesenchymal shape changing took place in the degenerated NP cells, whereas the AF cells showed no obvious changing between the two groups (Figure 1D). However, this shape change did not influence β‐actin expression (Figure S2C). Likewise, the NP cells from IDD tissues expressed a high level of miR‐640 (Figure 1E). In the AF cells, it did not show any significant changes (Figure 1F).

### 3.2 Inflammatory environment stimulated miR‐640 expression via NF‐κB signalling pathway

**Figure 2:**
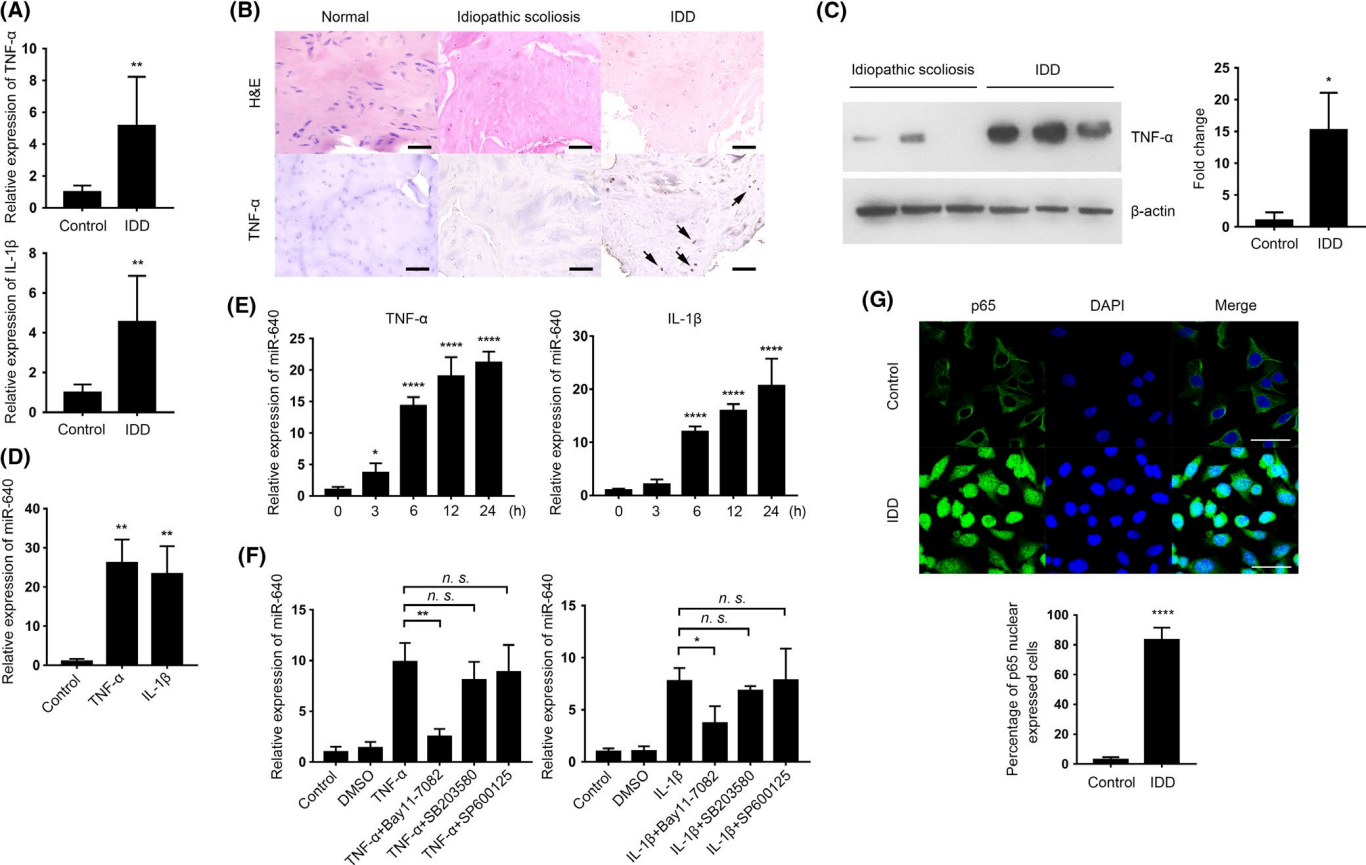
The proinflammatory cytokines promoted miR‐640 expression via NF‐κB signalling pathway. A, TNF‐α and IL‐1β mRNA levels in control and IDD‐derived NP cells were measured by qPCR. B, Different degenerated levels of IVD tissues were undergone H&E and immunohistochemical staining to exhibit TNF‐α expressing variation. Scar bar, 200 µm. C, Western blot analysis of TNF‐α expression in control and IDD tissues. D,E, miR‐640 expression in normal NP cells after TNF‐α or IL‐1β treatment for 1 d (D) and its accumulated process during 24 h (E). F, In TNF‐α/ IL‐1β and Bay11‐7082, SB203580, or SP600125 combinational treated NP cells, qPCR was performed to detect miR‐640 expression in each group. G, In normal and IDD‐derived NP cells, p65 expression was labelled by immunocytochemistry to identify the activation of NF‐κB signalling. The view field was randomly selected. Scar bar, 50 µm. Unpaired Student's *t* test and one‐way ANOVA were used to estimate the significant difference for all the qPCR data. n.s., not significant; *P ˂ .05; **P ˂ .01; ****P ˂ .0001

According to the close relationship between inflammation and degeneration,^8^ we detected the inflammatory cytokines TNF‐α and IL‐1β mRNA level in NP cells. In IDD‐derived NP cells, the levels of both cytokines were definitely elevated (Figure 2A). Moreover, TNF‐α protein level was also increased, especially in the high‐grade degenerative tissues (Figure 2B,C). These results indicated that the inflammatory environment was accompanied by IDD progression.

To investigate the relationship between inflammation and miR‐640 expression, we treated control NP cells with TNF‐α and IL‐1β. As shown in Figure 2D, the inflammatory cytokines induced a high level of miR‐640. And continuous stimulation of inflammatory circumstance led to miR‐640 accumulation (Figure 2E). However, the enhanced expression of miR‐640 did not have a TNF‐α or IL‐1β dose‐dependent. Low‐dose stimulation also effectively increased miR‐640 level (Figure S1). This suggested the TNF‐α and IL‐1β downstream pathways, such as NF‐κB, JNK and MAPK, might participate in the regulation of miR‐640 expression. To identify the key pathway which regulated miR‐640 expression, we treat the normal NP cells with various inhibitors: Bay11‐7082 (NF‐κB), SB203580 (MAPK) or SP600125 (JNK). Only NF‐κB signalling blockade significantly suppressed the TNF‐α, and IL‐1β induced high level of miR‐640 (Figure 2F). This result suggested that the inflammatory cytokines enhanced miR‐640 expression in NP cells via an NF‐κB dependent manner. Then, we performed immunofluorescence assay to confirm that in IDD‐derived NP cells, the NF‐κB signalling pathway was activated by the labelling of p65 nuclear translocation (Figure 2G).

**Figure 3:**
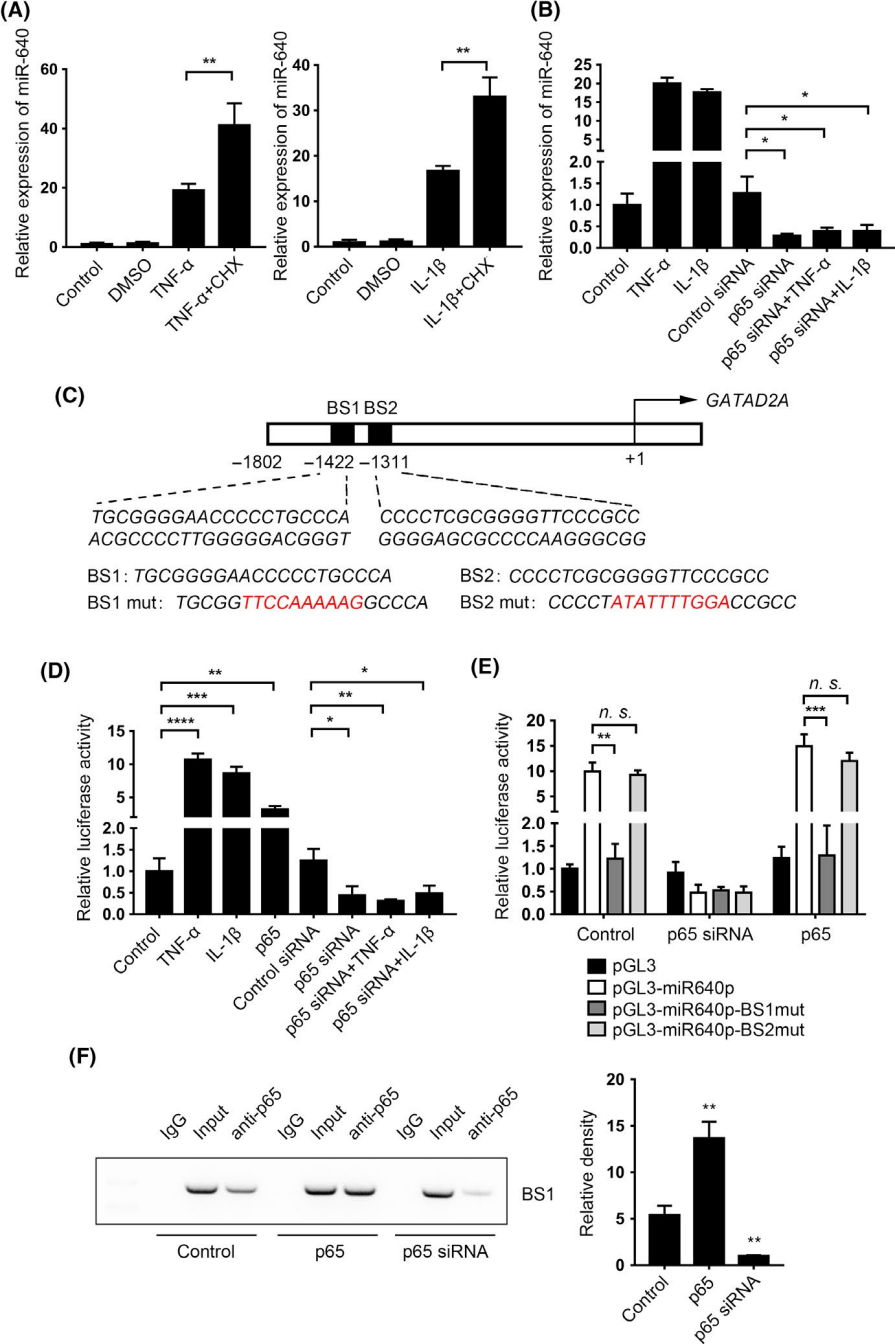
NF‐κB signal regulated miR‐640 transcription directly. A, The TNF‐α‐/ IL‐1β‐stimulated normal NP cells were pretreated with CHX, and the expression of miR‐640 was detected after 24 h by qPCR. B, miR‐640 expression was detected in the p65 knockdown NP cells, which were treated with TNF‐α or IL‐1β for 24 h. C, The schematic diagram of the miR‐640 promoter. Two predicted binding sites were marked. D, The relative luciferase activities were measured in 293T cells with the indicated treatment. E, Dual‐luciferase reporter system was used to estimate the binding activities between wild‐type/mutant miR‐640 promoter and p65 in 293T cells. F, In the 293T cells with the various transfected background, ChIP assay was performed to identify the physical bond of p65 and miR‐640 promoter. The histogram showed the grey value and statistic result, which was calculated by using the Mann‐Whitney test. One‐way ANOVA was used to evaluate the significant difference for the qPCR and luciferase reporter assay data. n.s., not significant; *P ˂ .05; **P ˂ .01; ***P ˂ .001; ****P ˂ .0001

Further, we detected whether miR‐640 is regulated directly by NF‐κB via cycloheximide (CHX) treatment, a protein synthesis inhibitor. Compared with TNF‐α or IL‐1β single treated cells, miR‐640 levels definitely increased after the combination with CHX (Figure 3A). Furthermore, the transfection of p65 siRNA (Figure S2A) reversed the accumulation of miR‐640 under TNF‐α and IL‐1β stimulations (Figure 3B). The results above strongly suggested NF‐κB regulated miR‐640 expression directly. Thus, we then analysed *GATAD2A* (where miR‐640 gene locates in) promoter and found out there were two p65 binding sites, which were provided by BioInformatics and Molecular Analysis Section (Figure 3C). Subsequently, we used the dual‐luciferase reporter system in HEK‐293T cells to verify NF‐κB directly regulated miR‐640 promoter activity. As shown in Figure 3D, TNF‐α, IL‐1β stimulation and p65 overexpression (Figure S3A) all could elevate luciferase activity. In contrast, after the p65 knockdown, the promoter activation was suppressed. To identify which binding site is responsible for NF‐κB signalling, we constructed two binding sites (BS) mutant plasmids. Coupled with TNF‐α treatment, the BS2 mutant group showed similar luciferase activity as the wide‐type group. However, the activity of BS1 mutant group significantly decreased when p65 presented (Figure 3E). This result indicated that BS1 is the central NF‐κB binding site in the miR‐640 promoter. Finally, via ChIP assay, we demonstrated that there was a direct binding between p65 and the promoter in TNF‐α‐treated NP cells. The quantities of precipitated promoter fragment were influenced by p65 expression level (Figure 3F). Taken together, all the results above suggested that the inflammatory environment in IDD tissues activated NF‐κB signalling pathway, which directly bound to miR‐640 promoter and enhanced its expression.

### 3.3 miR‐640 enhanced NF‐κB signal by LRP1 suppression

**Figure 4:**
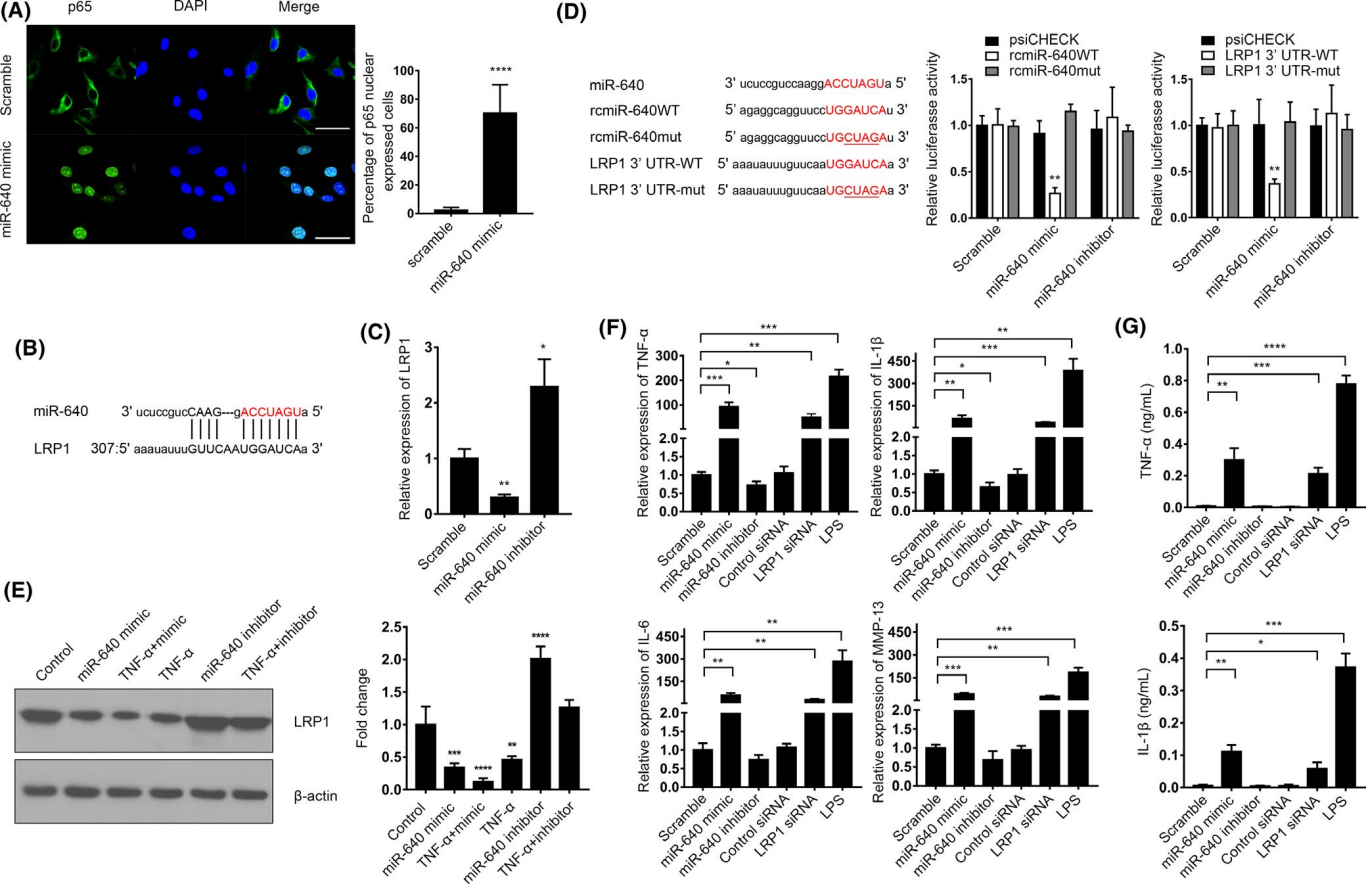
miR‐640 targeted to LRP1 to enhance NF‐κB signalling pathway activity. A, The immunocytochemistry assay was performed to reveal the location of p65 in miR‐640 overexpressed NP cells. The view field was randomly selected. Scar bar, 50 µm. B, The schematic diagram of miR‐640 targeted to *LRP1*. C, The mRNA levels of LRP1 in NP cells with or without miR‐640 expression were measured by qPCR. D, 293T cells were transfected with the indicated plasmids and miR‐640 mimic or inhibitor. Then, relative luciferase activities were measured to verify that miR‐640 directly bound to *LRP1* 3’UTR and promoted its degradation. E, LRP1 expression was detected by Western blot in the miR‐640 overexpressed or inhibited NP cells combined with 24 h of TNF‐α treatment. F, The mRNA expression of the NF‐κB signalling pathway target genes was detected by qPCR in NP cells with the indicated treatments. G, The ELISA assay was performed to measure the contents of the secreted TNF‐α and IL‐1β in the medium of NP cells with the indicated treatments. One‐way ANOVA was used to evaluate the significant difference for these data. *P ˂ .05; **P ˂ .01; ***P ˂ .001; ****P ˂ .0001

To reveal whether miR‐640 could influence NF‐κB signalling simultaneously, we found that after miR‐640 overexpression, p65 translocated into nuclei, which indicated NF‐κB signalling was activated (Figure 4A). To illustrate the mechanism involved in this reverse regulation, we noticed that *LRP1*, a strong indirect inhibitor of NF‐κB,^26^ might be a potential target gene of miR‐640 (Figure 4B). We then detected miR‐640 levels in the normal NP cells. The result consisted with the previous prediction, miR‐640 inhibited LRP1 expression (Figure 4C). As shown in Figure 4D, we built the plasmids, which were inserted the listed sequence, and demonstrated that miR‐640 bound to wild‐type *LRP1* 3’‐UTR and significantly decreased its luciferase activities. In addition, we also measured the LRP1 protein levels in normal NP cells. TNF‐α and miR‐640 could suppress LRP1 expression definitely. Furthermore, the TNF‐α‐associated LRP1 reduction was miR‐640 dependent (Figure 4E). All these data indicated that miR‐640 targeted LRP1.

Finally, the mRNA expression of NF‐κB target genes was detected by qPCR. miR‐640 overexpression and LRP1 knockdown (Figure S2B) all promoted these genes transcription (Figure 4F). LPS was set as a positive control. Similarly, the protein levels of TNF‐α and IL‐1β elevated after miR‐640 transfection or LRP1 silencing (Figure 4G). All the results above suggested that the inflammatory cytokines induced a high level of miR‐640 enhanced the production of TNF‐α and IL‐1β, which composed a positive feedback loop to aggravate the inflammation in IVD. It also suggested that miR‐640 played an indispensable role in the regulation of NF‐κB signalling in NP cells.

### 3.4 miR‐640 inhibited WNT signalling via the regulation of β‐catenin and EP300 expression

**Figure 5:**
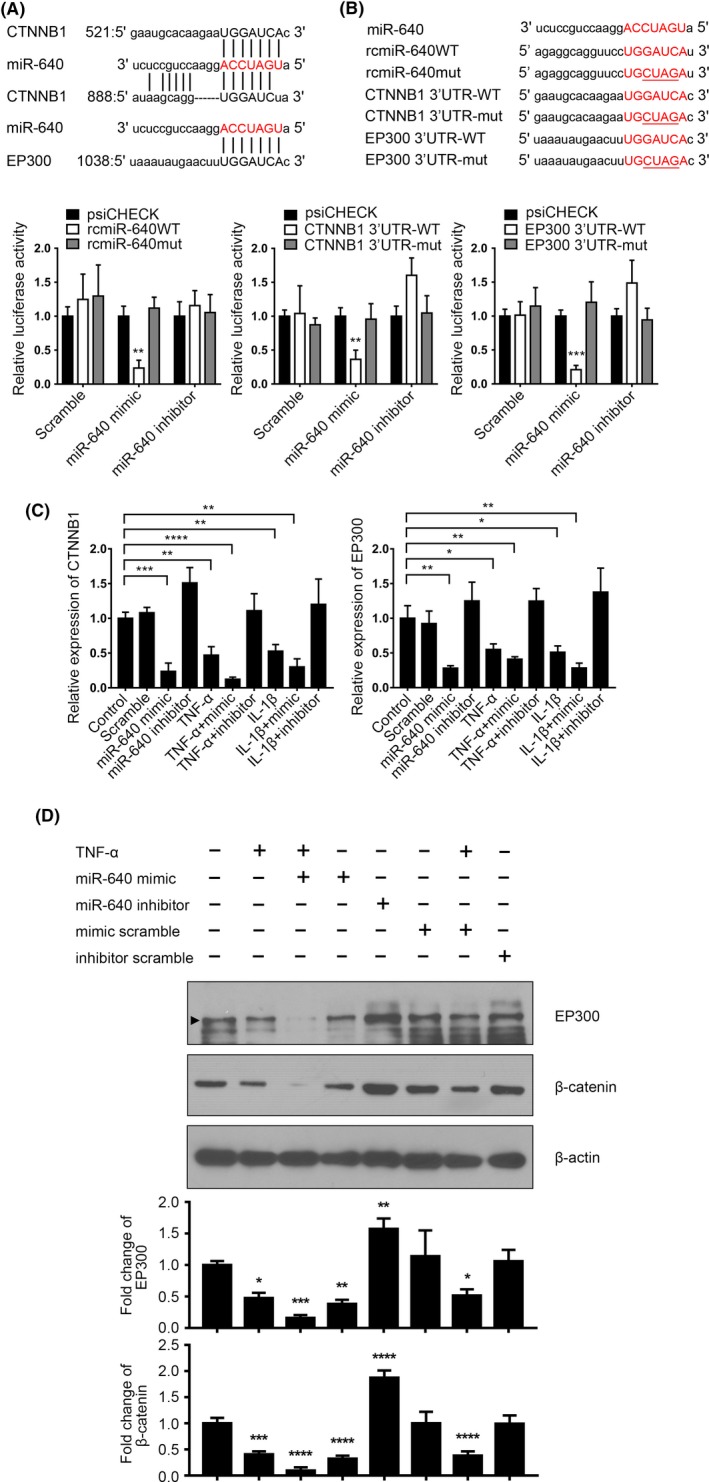
miR‐640 inhibited β‐catenin and EP300 expression. A, The schematic diagram of miR‐640 targeted to *CTNNB1* and *EP300*. B, 293T cells were transfected with the indicated plasmids and miR‐640 mimic or inhibitor. Then, relative luciferase activities were measured to verify that miR‐640 directly bound to *CTNNB1* and *EP300* 3’UTR and promoted its degradation. C,D, The expression of β‐catenin and EP300 was detected by qPCR (C) and Western blot (D) in the TNF‐α‐/ IL‐1β‐stimulated NP cells with or without miR‐640 presence. One‐way ANOVA was used to evaluate the significant difference for these data. *P ˂ .05; **P ˂ .01; ***P ˂ .001; ****P ˂ .0001

We had demonstrated that miR‐640 exacerbated inflammatory response. However, the functions of miR‐640 in IVD degeneration were still not clear. Based on bioinformatics analysis, *CTNNB1* and *EP300*, two important members of the WNT signalling pathway, were picked out as the potential targets of miR‐640 (Figure 5A). As shown in Figure 5B, we confirmed that miR‐640 could inhibit luciferase activities of wild‐type *CTNNB1* and *EP300* 3’UTR. miR‐640, as well as TNF‐α and IL‐1β, inhibited *CTNNB1* and *EP300* expression in NP cells (Figure 5C). The proinflammatory cytokine‐induced suppression of the target genes was miR‐640 dependent, due to the miR‐640 inhibitor transfected NP cells under TNF‐α or IL‐1β treatment did not show any significant changes of the indicated genes. Likewise, the protein levels of β‐catenin and EP300 all suppressed by miR‐640 and TNF‐α, which could be rescued by the inhibitor transfection (Figure 5D).

**Figure 6:**
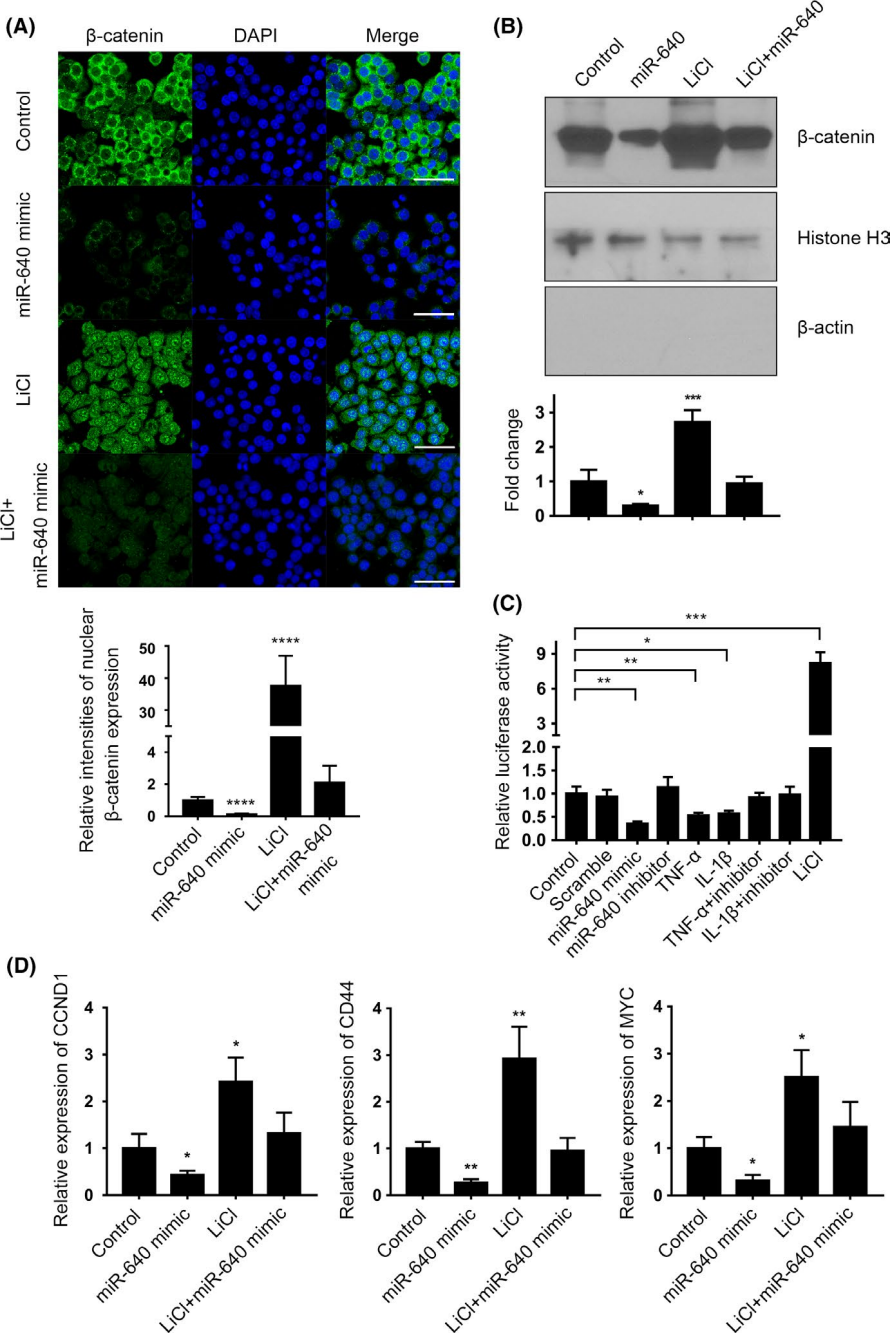
miR‐640 repressed WNT signalling pathway. A, The immunocytochemistry assay was performed to reveal the expression and location of β‐catenin in miR‐640 overexpressed and/or LiCl‐stimulated NP cells. The view field was randomly selected. Scar bar, 50 µm. B, The nuclear expression of β‐catenin was estimated by Western blot in miR‐640 overexpressed and/or LiCl‐stimulated NP cells. Histone H3 was set as a nuclear marker, and β‐actin was a cytoplasmic marker. C, The TopFlash luciferase reporter system was used to assess WNT signalling pathway activities in TNF‐α/IL‐1β‐stimulated 293T cells with different miR‐640 expression level. D, The mRNA level of the WNT signalling pathway target genes was measured by qPCR in miR‐640 overexpressed and LiCl‐stimulated NP cells. One‐way ANOVA was used to evaluate the significant difference. *P ˂ .05; **P ˂ .01; ***P ˂ .001; ****P ˂ .0001

The hallmark event of WNT signalling pathway activation is that β‐catenin escapes from the arresting of GSK‐3β, Axin, APC and WTX, and moves into nuclear to regulate transcription.^27^ 10 mmol/L LiCl was used to simulate WNT activation,^28^ which was attenuated by miR‐640 overexpression in NP cells (Figure 6A). The Western blot assay showed a similar result that miR‐640 reduced the β‐catenin expression level within the nuclei, even after LiCl stimulation (Figure 6B). To verify miR‐640’s regulatory effects of the integral WNT signalling pathway, we performed the luciferase reporter assay by using the TopFlash system, which was used to measure β‐catenin‐driven TCF/LEF transcriptional activation.^29^ miR‐640 definitely inhibited WNT signalling, and similarly, the repression induced by TNF‐α and IL‐1β was miR‐640 dependent (Figure 6C). All these results indicated that miR‐640 was a negative regulator of WNT signalling in NP cells. Thus, the expressions of the downstream genes of β‐catenin, such as *CCND1*, *CD44* and *MYC*, should be inhibited by miR‐640, which consisted with our observation (Figure 6D).

### 3.5 miR‐640 induced NP cell degeneration

**Figure 7:**
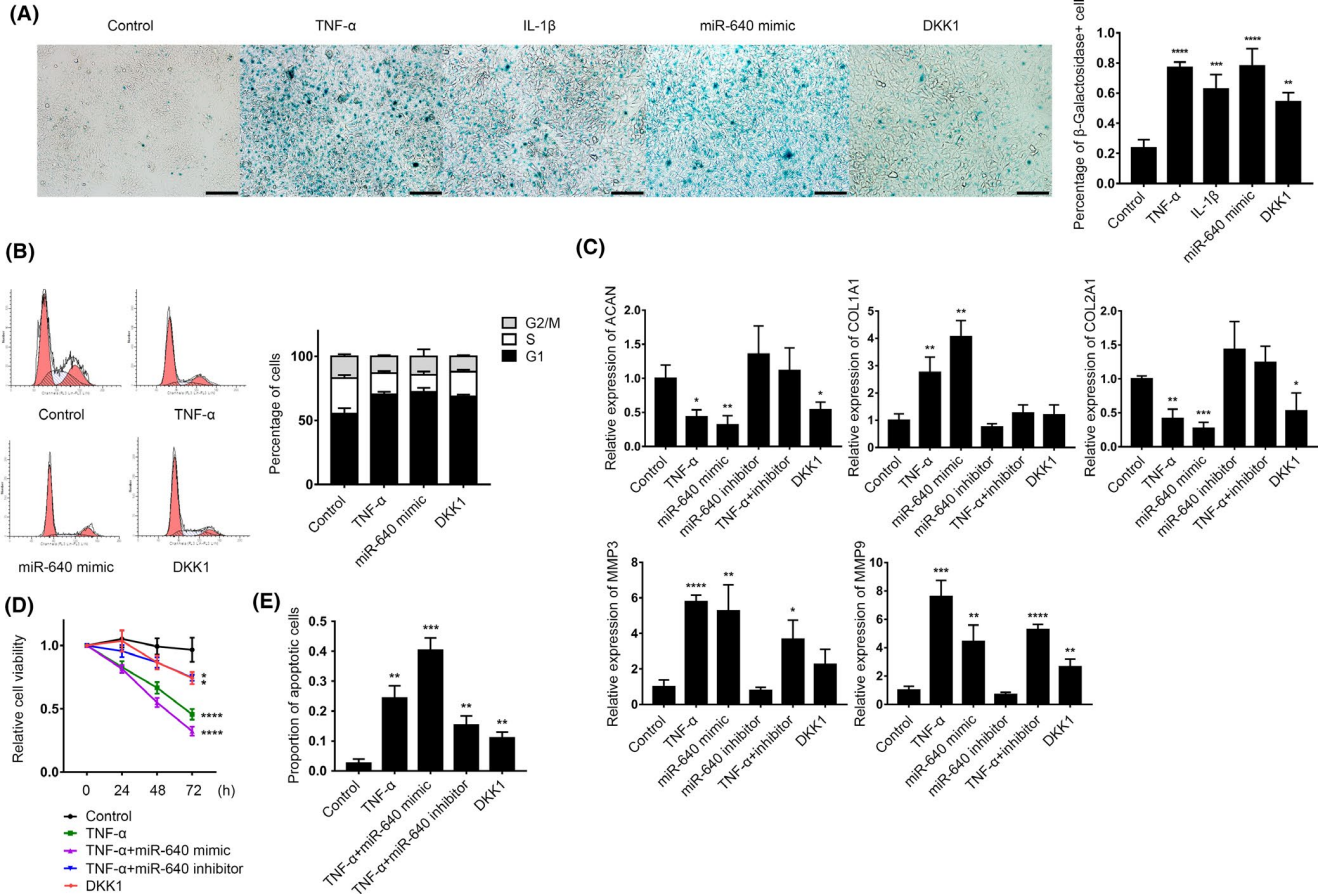
miR‐640 promoted NP cells degeneration. A, β‐galactosidase staining was used to label the senescence NP cells with TNF‐α, IL‐1β, DKK1 treatments for 24 h and miR‐640 overexpression. The view field was randomly selected. Scar bar, 200 µm. B, Flow cytometry was used to analyse cell cycle distribution of the TNF‐α, DKK1 treatments and miR‐640 overexpressed NP cells. In the flow cytometry plots, the left peak is G1 phase, and the right peak is G2 phase. The slash area between the two peaks indicates S phase. C, The mRNA levels of several IDD‐associated genes were measured by qPCR in the NP cells with the indicated treatments. D, ATP consumption‐based cell viability detection assay was performed to assess the different treated NP cells vitality. E, The TNF‐α‐ and miR‐640‐induced NP cell apoptosis was assessed by flow cytometry. The statistical analysis was achieved by using Mann‐Whitney test. One‐way ANOVA was used to evaluate the significant difference for the other data. *P ˂ .05; **P ˂ .01; ***P ˂ .001; ****P ˂ .0001

Because of the essential functions of WNT signalling during senescence and apoptosis, miR‐640 was reasonably predicted as a key regulator in IDD. After proinflammatory cytokine treatment and miR‐640 overexpression in normal NP cells, the β‐galactosidase staining positive cells, a marker of cell senescence, dramatically increased (Figure 7A). DKK1 is a constitutive inhibitor of WNT signalling, which was set as a positive control. The cell cycle analysis showed that WNT signalling repression induced cell cycle blockade in G1 phase, another hallmark of cell senescence (Figure 7B). We then quantified the expression of several IDD‐associated marker. Not surprisingly, the level of IDD‐accompanying genes, *COL1A1*, *MMP‐3* and *MMP‐9,* significantly increased based on TNF‐α stimulation and miR‐640 transfection, whereas *ACAN* and *COL2A1* decreased (Figure 7C). Remarkably, the changes of ACAN, COL1A1 and COL2A1 could be attenuated by miR‐640 inhibitor transfection, which indicated their expressions were mainly regulated by WNT signalling. However, the inhibitor could not completely neutralize the effects of TNF‐α on *MMP‐3* and *MMP‐9*. This suggested the two genes were not only regulated by inflammation‐associated WNT repression. All these results indicated that the inflammatory environment repressed NP cells WNT signalling pathway and promoted their degeneration. miR‐640 inhibitor treatment could relieve the effects to some extent.

Apoptosis is another trait of NP cell degeneration. The ATP consumption‐based cell viability analysis showed that inflammation dramatically reduced NP cells vitality. miR‐640 inhibitor transfection could bring partial remission (Figure 7D). Likewise, TNF‐α treatment was accompanied by a large number of apoptotic cells, which could also be neutralized by miR‐640 inhibition (Figure 7E, Figure S3). All the results above indicate that inflammation and the following miR‐640‐associated WNT signalling repression cause NP cells degeneration. miR‐640 sequestering showed an impressive therapeutic effect to oppose IDD.

## 4 DISCUSSION

Recent studies have unveiled the important role of inflammation in IDD from in vitro,^12^ in vivo,^30^ and clinical trail^31^ levels. In bone or cardiovascular tissues, the anti‐inflammation treatments have been verified to be critical in tissues repair and regeneration. However, the effects of inflammation control are not clear in IVD tissue.^32^ This may partly due to the complicated regulatory and feedback effects of multiple inflammation downstream signalling pathways and their interactions.^9^ The specific intra‐ or extracellular environment of the IVD may also contribute to the complexity. For example, Nava et al^33^ showed that TNF‐α promoted WNT activity and β‐catenin nuclear translocation in an IFN‐r‐ and PI3K‐AKT‐dependent fashion in intestinal epithelial cells. Jang et al^34^ found TNF‐α activated WNT/β‐catenin pathway in BEAS‐2B human bronchial epithelial cells. But in other tissues, the TNF‐a‐related NF‐kB and WNT signalling pathways were always competing.^35^, ^36^ Especially in bone, TNF‐a took the regulation of Semaphorin3B from WNT signalling pathway, which ultimately induced osteoporosis.^37^ The effects of the NF‐kB and WNT signalling pathways interactions are likely to depend on the specific cell or tissue type. Furthermore, β‐catenin is not only an intracellular signal transductor but also an important cell adhesion factor in the epithelial cell. It is critical in cells tight junction and epithelial outline maintenance when it interacts with E‐cadherin.^38^ In our study, we found during the development of IDD, β‐catenin was downregulated by TNF‐α‐induced miR‐640 expression. In another hand, the IDD‐derived NP cells showed a typical mesenchymal appearance. The relationship between miR‐640 and EMT may be an additional mechanism in IDD development. But it still needs to be uncovered. Therefore, we need to illuminate the molecular mechanisms of IDD development comprehensively. This will help us to identify effective drug targets.

At present, the clinical treatment for IDD mainly includes conservative treatment and surgery. Conservative treatment is divided into drug and physical treatment. Although conservative treatment alleviates the IDD‐derived torment, it cannot influence the degenerated process and eradicate the source of the disease. Surgical treatment can directly address the cause and relieve spinal cord compression. However, it also causes many other complications. As a new choice, biotherapy has been widely used in clinical practices. Some TGF‐β family members^39^, ^40^, ^41^ and PDGF^42^ showed anti‐IDD effects in vitro and in vivo. All these studies demonstrate that biotherapy has great prospects of the IDD treatment. However, the low stability and side effects of the macromolecules, the safety and efficiency of administration, and the individual difference limited those potential drugs to use in clinical trial. In recent years, some studies showed that microRNAs functioned in many aspects of IDD. Wang HQ et al^25^ showed that in IDD cells, low miR‐155 level induced Fas‐associated cell apoptosis. Liu G et al^43^ demonstrated that miR‐27a inhibited PI3K‐Akt signalling pathway and activated caspase‐3 to promote NP cell apoptosis. The reduction of miR‐129‐5p unleashed its targets *COL1A1* and *ITGA1* expression, which promoted IDD.^44^ Compared to proteins, microRNA should decrease the frequency of hazard and side effects, because it does not need to be transfected into target cells. The low molecular weight of microRNA brings some advantages in drug delivery and uptake efficiency. Taken together, microRNA is a promising candidate for future IDD biotherapy.

In this study, based on the screening result of microRNA array, we found miR‐640 overexpressed in IDD NP cells. High levels of TNF‐α and IL‐1β promoted its expression through NF‐κB signalling pathway. A positive feedback loop, which involved NF‐κB signalling and miR‐640, exacerbated inflammation and miR‐640 accumulation. Eventually, miR‐640 targeted to WNT signalling pathway and induced NP cell degeneration. Our work clarified the mechanism of inflammation‐induced IDD progression and the specific role of miR‐640 in this regulatory network. However, the impacts of protein levels of β‐catenin and EP300 cannot fully explain the WNT signalling pathway activation. Other functions of miR‐640 in IDD still need to be further understood to avoid potential side effects. In vivo data of anti‐miR‐640 treatment were also required to evaluate the therapeutic effects and the efficiency of drug delivery.
