# Supplementary figures and images for: miR‐640 aggravates intervertebral disc degeneration via NF‐κB and WNT signalling pathway
Source: Cell Prolif. 2019 Jul 25;52(5):e12664. doi: 10.1111/cpr.12664 (PMC6797513; doi:10.1111/cpr.12664)

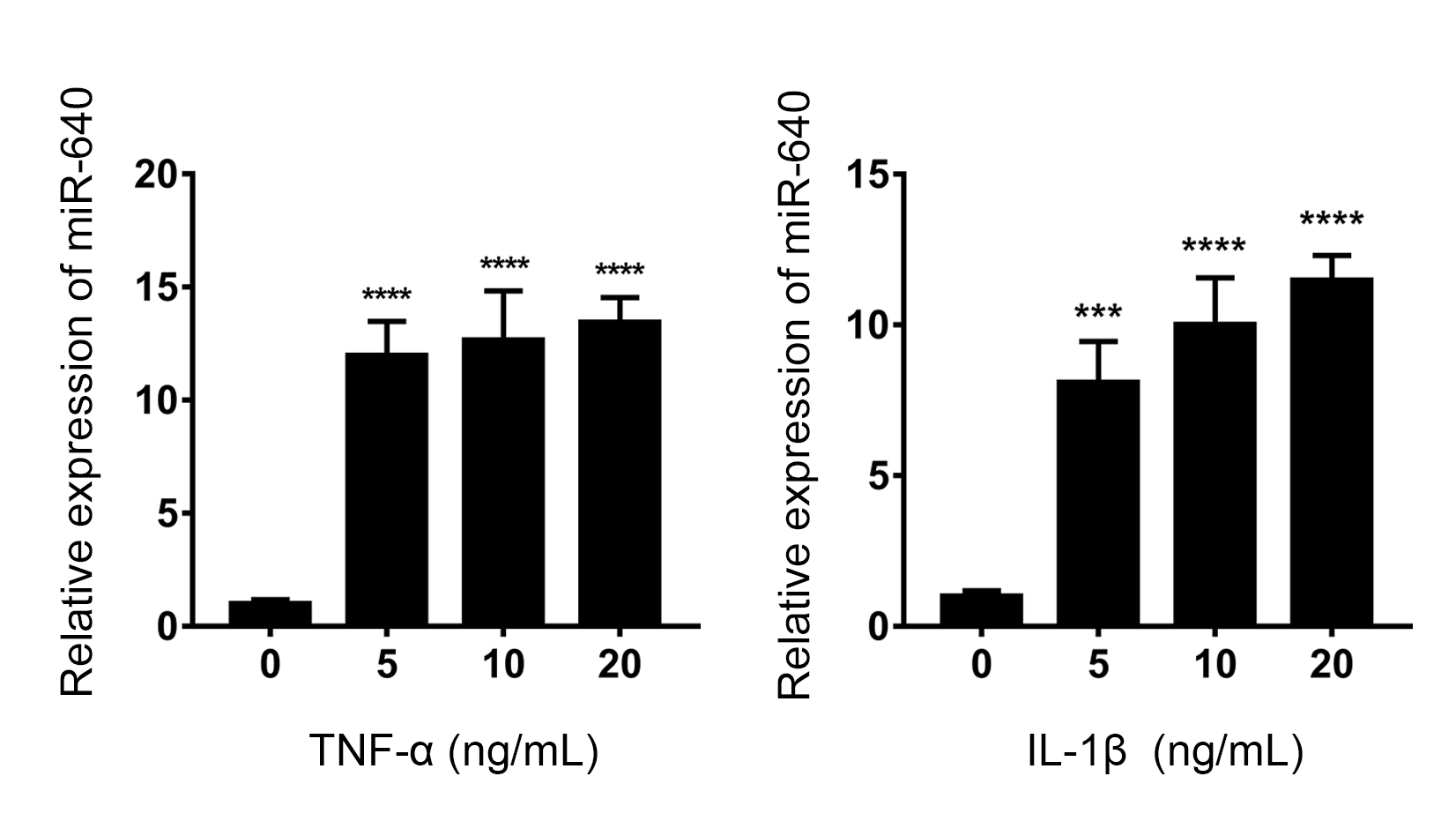

Supplement: Supplementary file 1 [file CPR-52-e12664-s001.tif]

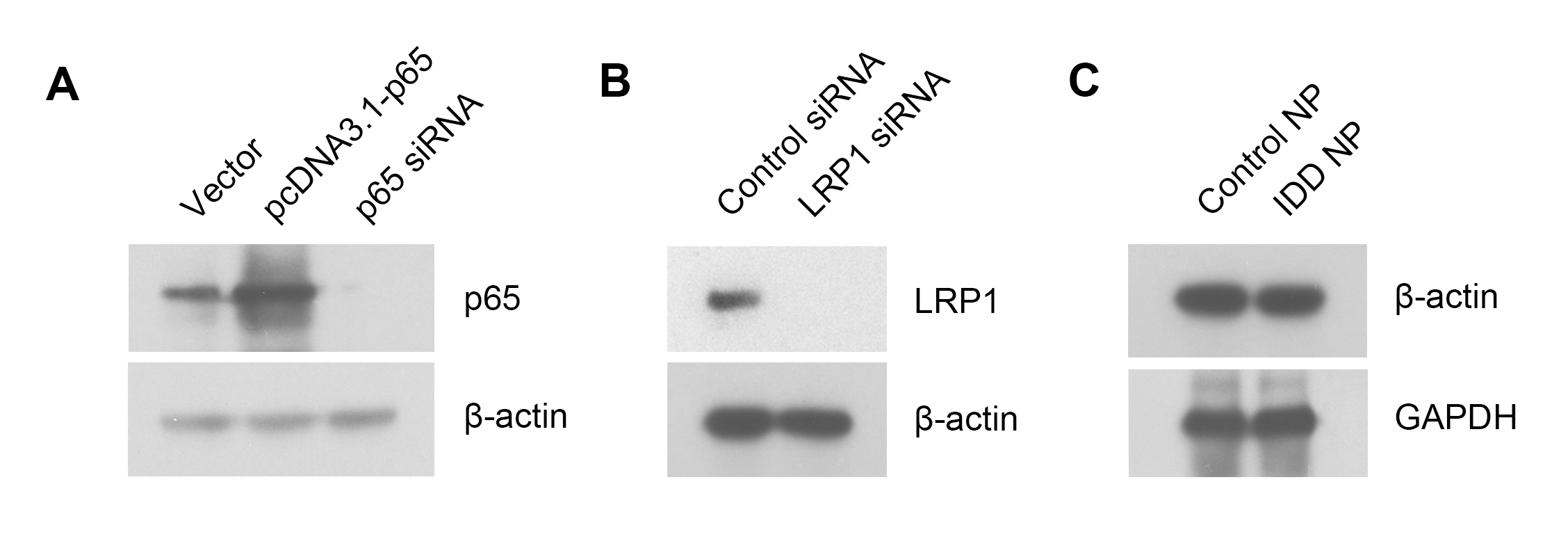

Supplement: Supplementary file 2 [file CPR-52-e12664-s002.tif]

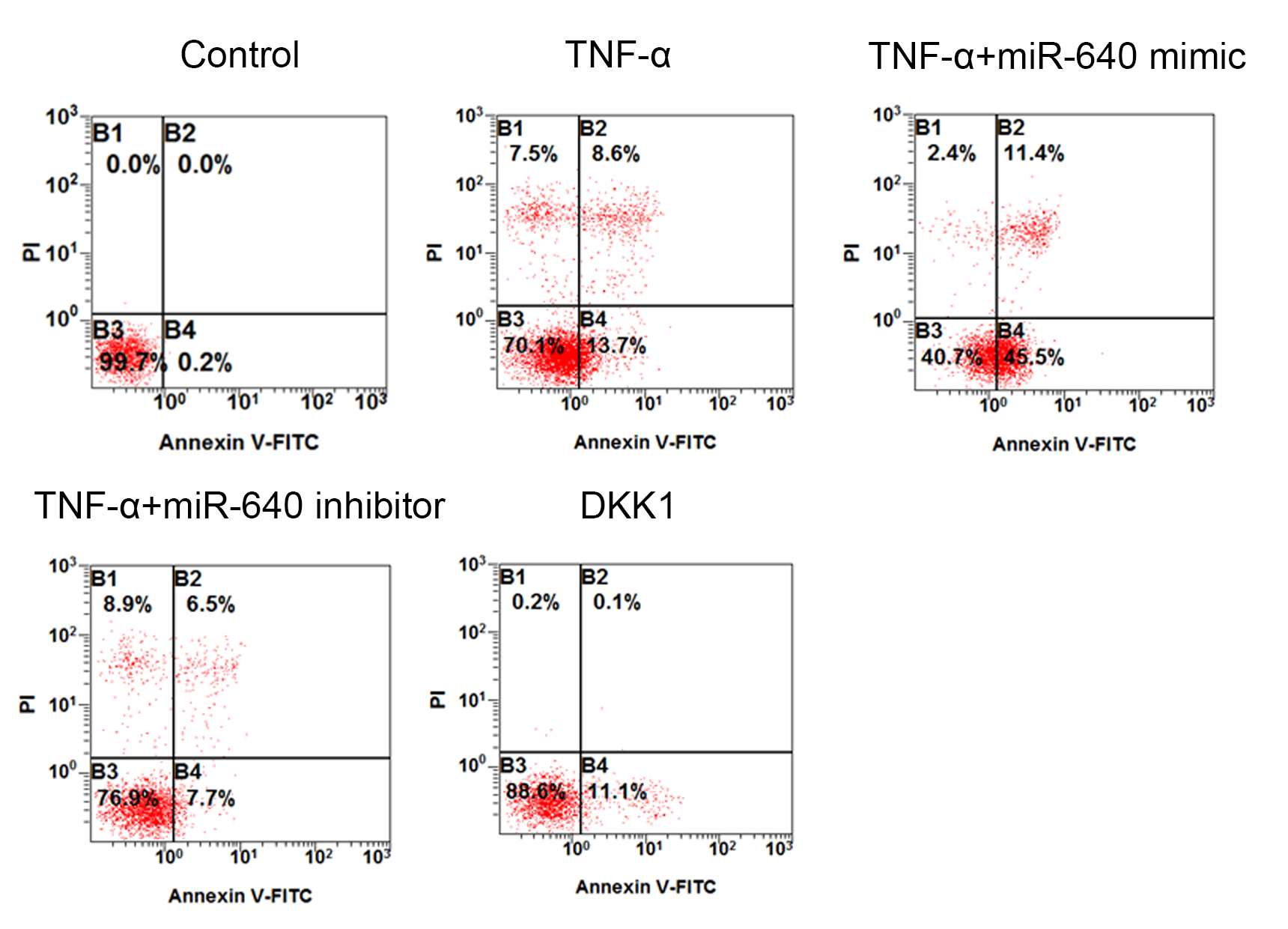

Supplement: Supplementary file 3 [file CPR-52-e12664-s003.tif]
